# Titanium Base Abutments in Implant Prosthodontics: A Literature Review

**DOI:** 10.1055/s-0041-1735423

**Published:** 2021-11-18

**Authors:** Ahmad M. Al-Thobity

**Affiliations:** 1 Department of Substitutive Dental Sciences, College of Dentistry, Imam Abdulrahman Bin Faisal University, Dammam, Saudi Arabia

**Keywords:** abutment, review, titanium base, titanium insert

## Abstract

Implant abutments are essential components in restoring dental implants. Titanium base abutments were introduced to overcome issues related to existing abutments, such as the unesthetic appearance of titanium abutments and the low fracture strength of ceramic abutments. This study aimed to comprehensively review studies addressing the mechanical and clinical behaviors of titanium base abutments. A search was performed on PubMed/MEDLINE, Web of Science, Google Scholar, and Scopus databases to find articles that were published in English until December 2020 and that addressed the review purpose. A total of 33 articles fulfilled the inclusion criteria and were included for data extraction and review. *In vitro* studies showed that titanium base abutments had high fracture strength, adequate retention values, particularly with resin cement, and good marginal and internal fit. Although the clinical assessment of titanium base abutments was limited, they showed comparable performance with conventional abutments in short-term evaluation, especially in the anterior and premolar areas. Titanium base abutments can be considered a feasible treatment option for restoring dental implants, but long-term clinical studies are required for a better assessment.

## Introduction

Osseointegrated dental implants have been proven to be an ideal treatment modality in restoring the oral function and esthetic of missing teeth because of their clinical survival rates. 1 2 3 The prosthetic components of dental implants have been developed dramatically to secure biocompatibility, harmonize the adjacent soft and hard tissues, and improve the esthetic and biomechanical merits. 4 Implant abutments are used to connect the implant body with implant-supported restorations. Numerous materials and techniques have been conducted to fabricate implant abutments based on different clinical situations. 5 6 7

Prefabricated titanium abutments are the most common type used because they have a simple technique and are inexpensive compared with other types. 8 9 However, these abutments may only be applicable to cement-retained restorations, cases with ideal implant placement, and cases that suit the depth, emergence profile, and diameter of the restored edentulous area. 10 11 Custom abutments have been suggested to overcome the disadvantages of prefabricated abutments, particularly in off-axial implants in which screw access emerges buccally. Custom abutments can either be cast using metal alloys or milled by computer-aided design/computer-aided manufacturing (CAD/CAM) technology. They provide high strength, long durability, and either cement- or screw-retained prosthesis, and they allow the fabrication of a fixed prosthesis with proper thickness. 12 Despite their advantageous properties, these abutments have limited application due to their fabrication sensitivity, high price, and inappropriate esthetic appearance. 13 14

Dentists’ attention has turned toward ceramic abutments to fill the need for suitable abutments in the esthetic zone. Owing to their adequate biomechanical and optical properties, zirconia abutments have been commonly used in either cement- or screw-retained implant-supported prostheses. 15 16 17 18 These abutments can be offered in a one-piece design made of zirconium oxide, including the abutment and the internal connection part, or a two-piece design in which a metallic insert is included as an internal connection. 19 20 In a 10-year randomized prospective study, Amorfini et al 15 investigated the clinical outcomes of one-piece and two-piece zirconia abutments and found that the overall prosthetic success rate was 85% and that the observed prosthetic complications included abutment fracture, porcelain chipping, screw loosening, and loss of retention. A 12-year retrospective study reported similar complications related to zirconia abutments, such as abutment fracture occurring at the implant neck and along the abutment walls adjacent to the screw access hole. 21 Stimmelmayr et al 22 investigated the wear at the abutment implant interface with zirconia and titanium abutments and found that a significant higher wear of titanium implants was noticed when connected to one-piece zirconia abutments.

Recently, the use of a digital workflow through CAD/CAM systems has been developed in implant dentistry to allow the precise machining of implant-supported prostheses in a shorter duration. 23 Thus, titanium base abutments were introduced to allow for a strong link between the implant and the ceramic abutment/crown and to provide a favorable esthetic outcome. 24 This review aimed to focus on the technical and clinical applications of titanium base abutments in implant prosthodontics. Particular attention was given to the titanium base abutment design, surface treatment and retention of the superstructure, fracture strength and failure mode, misfit and torque loss, and clinical performance of titanium base abutments.

## Methods

A comprehensive search was conducted on PubMed/MEDLINE, Web of Science, Google Scholar, and Scopus databases until December 2020 to identify *in vitro* and *in vivo* studies addressing the mechanical properties and clinical performance of titanium base abutments. The search keywords included titanium base abutment, titanium base, titanium insert, and low-profile titanium abutment. Title and abstract reviews were performed to identify the articles that met the review objectives. A total of 33 studies were included for data extraction and review under the following categories: titanium base design, surface treatment and retention of superstructure, fracture strength and failure mode, misfit and torque loss, and clinical performance.

## Results

### Titanium Base Design

Titanium base abutments have a specific geometry that is saved in the CAD/CAM system to allow for the fast fabrication of restorations. Once the restoration is milled and has undergone sintering or the crystallization cycle, it is cemented or bonded to the titanium base extraorally and then inserted into the dental implant. 11

Two techniques are used to fabricate implant-supported restorations using titanium base abutments. 11 25 26 27 The first technique is to design and mill the crown and abutment as one piece using CAD/CAM ceramic restorations or create a wax up using a plastic sleeve and fabricate the restoration using the pressable ceramic materials. After that, the crown can be bonded to the titanium base abutment. The advantage of this technique is that it removes excess cement extraorally before the abutment is screwed into the implant. 11 25 28 The second technique involves designing and milling, or pressing the abutment and the crown separately, followed by bonding the abutment to the titanium base. The abutment is then screwed into the dental implant, followed by crown cementation on the abutment. 26 27 Nouh et al 26 assessed the fracture resistance of these two techniques using zirconia and lithium disilicate restorative materials and found that the abutment bonded to the titanium base with a separate zirconia crown had the highest fracture resistance (3,730 N), followed by the one-piece zirconia abutment and crown bonded to the titanium base (3,400 N), with no significant difference between both techniques.

Recently, titanium base abutments with the concept of angled screw channel have been manufactured to compensate for the buccal/labial angulated implant position. 29 30 31 The benefit of this concept is to allow for fabrication of screw-retained restorations by redirecting the screw access channel to the lingual aspect. The corrected angulation of these abutments ranges between 0 and 30 degrees to the long axis of the implants. 29 A specific hexalobular head design of the abutment screws has been fabricated to allow engaging of a specific screwdriver to tightening and torquing the screw.

The height of titanium base abutments varies based on the available restorative space. 25 26 Silva et al 25 evaluated the effect of two different heights of titanium base abutments (4 and 2.5 mm) on the retention of zirconia crowns using the pull-out test in a universal testing machine. They reported no significant effect of the abutment height on the retention of the crown.

### Surface Treatment and Retention of Superstructure

Different cement materials, cementation techniques, and surface treatment procedures have been investigated in *in vitro* models to assess the pull-out retention strength between the titanium base abutments and the superstructures of either abutments or crowns. 25 32 33 34 35 Three types of cements, including temporary cement, resin cement, and glass ionomer cement, have been tested for the tensile bond strength test between titanium base abutments and zirconia copings. Resin cement presented a significant increase in retention values compared with temporary cement and glass ionomer cement. 25 In this study, both the titanium base and zirconia superstructure were treated with an adhesive system, and no mechanical surface treatment methods were used. 25 In another *in vitro* study, temporary cement and self-adhesive resin cement were used to evaluate the retention of four superstructure materials to titanium base abutments. 35 A substantial difference in retention values was reported between the two cements, with resin cement having the highest retention mean value.

Gehrke et al 34 examined the effectiveness of three resin cements in retaining zirconia copings to titanium base abutments. All titanium base abutments and zirconia copings were subjected to air abrasion using 50 µm aluminum oxide particles and 15,000 cycles of thermocycling. Although the retention values of the three cements were high enough to provide stable retention, the difference between the cements was not significant. 34 In another study, three different resin cements were used to evaluate the retention of zirconia and lithium disilicate copings to titanium base abutments. 32 Different mechanical and chemical surface treatments, such as sandblasting with 50 µm aluminum oxide particles and bonding agents, were applied to the surface of titanium base abutments and the inner surface of ceramic copings. The results showed that the combination of chemical and mechanical surface treatments significantly enhanced the retention of lithium disilicate and zirconia copings, regardless of the cement type. 32

Therefore, it is recommended to modify the surfaces of titanium base abutments and superstructure materials with chemical and mechanical surface treatments to improve joint retention. Resin cement is the preferred luting agent to cement the two components together.

### Fracture Strength and Failure Mode

Although the fabrication of abutments completely using zirconia has improved the esthetic outcomes, particularly in the esthetic zone, these abutments demonstrate a weak connection and are vulnerable to fracture. 36 37 38 One of the main advantages of using titanium base abutments is the improved fracture resistance of the ceramic abutments and crowns, thus overcoming the brittle nature of ceramic abutments. 24

Several studies investigated the effect of introducing titanium base abutments into implant-supported restorations. 24 27 39 40 41 42 43 44 45 Different designs of zirconia abutments, including one-piece anatomic contour zirconia abutments and zirconia abutments with titanium inserts, have been examined for fracture strength tests after screwing them to titanium alloy implants with a regular diameter (4.1 mm). 24 Zirconia abutments with titanium inserts were found to have a remarkable increase in fracture resistance compared with the one-piece zirconia. The fracture of one-piece anatomic contour zirconia abutments occurred either at the coronal part of the abutments or at the hexagon connection part. By contrast, neither the zirconia abutments nor the titanium inserts had fracture in the zirconia abutments with titanium inserts; the fracture occurred only in the abutment screws. 24 However, the one-piece zirconia abutments should be used with caution in the posterior segments, as the average recorded value of occlusal forces posteriorly could increase to 720 N. 24 46

Elsayed et al 27 compared the fracture strength of different types of abutments, including titanium, zirconia, zirconia with titanium inserts, lithium disilicate abutments with titanium inserts, and combined lithium disilicate abutments and crowns with titanium inserts. All abutments were restored with lithium disilicate crowns and screwed to titanium implants with a regular diameter. The authors reported that the lowest fracture resistance value was found in the one-piece zirconia abutments, with the fracture occurring at or above the implant shoulder level. The other abutment types with titanium inserts had significantly higher fracture resistance values, and failure occurred because of the bending of the titanium inserts and screws. 27

Regarding screw-retained implant-supported restorations, a recent study evaluated the fracture strength of partially stabilized and fully stabilized monolithic zirconia crowns screwed directly to implants or cemented to titanium base abutments. 40 The results showed that the screw-retained monolithic zirconia crowns with titanium base abutments either partially stabilized or fully stabilized were significantly stronger than the screw-retained zirconia crowns without a titanium base. 40 In another study, lithium disilicate, zirconia, and polyetheretherketone materials were employed to fabricate screw-retained implant-supported single crowns (combination of abutments and crowns) using titanium base abutments, and their fracture resistance was investigated. Zirconia crowns with titanium base were found to have higher fracture resistance than other materials, and they could be used in the premolar area. 45 Adolfi et al 44 assessed the fracture resistance of two different designs of assembling screw-retained zirconia crowns to titanium bases. In the first design, the titanium bases were cemented to the zirconia crowns using resin cement; in the second design, the zirconia crowns were fixed to titanium bases through a hexagonal connection notched in both the crowns and titanium bases. The authors reported that the group with titanium bases cemented to zirconia crowns had a significantly greater fracture load than the notched restorations. They concluded that the resin cement applied between the restoration and the titanium base could have the potential to improve fracture resistance. 44

Based on the results of previous studies, implant-supported ceramic restorations should be braced using titanium base abutments to withstand occlusal forces due to high bending moments. 24

### Misfit and Torque Loss

One of the main requirements to achieve a successful implant-supported restoration is for the implant to passively fit. 47 48 The misfit can induce stresses to the implant–bone interface and create biological and mechanical complications, such as torque loss and screw loosening, fracture of abutment screw, marginal bone loss around the implant neck, and loss of implant osseointegration in advance cases. 49 50 Previous studies have suggested that a 150-µm gap can be considered a clinically acceptable misfit value. 51 52

Many attempts have been conducted to explore the effect of using titanium base abutments on the misfit of implant-supported restorations. 40 44 53 54 55 Ramalho et al 54 assessed the internal fit of implant-supported single crowns fabricated from different designs, including three screw-retained restorations (milled one-piece abutment/crown, milled crown cemented to a titanium base, and milled crown cemented to custom abutments) and three cement-retained restorations (milled two-piece abutment and crown, milled crown cemented to a titanium base, and milled crown cemented to custom abutments). They found that restorations with a titanium base and custom abutments had significantly lower misfit values than digitally milled restorations. Similarly, in another study, fully digital, titanium base, and custom abutments were fabricated and assessed for internal fit in different regions of the implant abutment connection (marginal, top, and middle of the connection) using the silicon replica technique and microcomputed tomography. 55 Titanium base and custom abutments were found to have a better internal fit than digitally milled abutments. 55

A recent study assessed the misfit of screw-retained single-unit restorations constructed by milling, titanium base, casting, overcasting, and laser sintering processing methods. 53 Titanium base abutments were found to have a significantly better marginal fit than the casting and laser sintering techniques and a lower fit than the milling process method. All fabrication techniques showed a misfit of restorations less than 150 µm. 53

Regarding torque loss, Adolfi et al 44 compared the amount of torque loss, vertical misfit, and stress concentration between zirconia restorations after being cemented to titanium base abutments using resin cement or notched to a titanium base using the hexagon shape of the inner surface of zirconia crowns and the outer surface of the titanium base. The authors reported that the amount of torque loss, stress concentration, and vertical misfit decreased significantly in the cement-retained restorations compared with the notched-retained restorations. 44 In a recent study, the amount of torque loss of titanium bases was evaluated after being bonded to zirconia, lithium disilicate, or polyetheretherketone restorations, 45 and the material of the superstructure was found to have no significant effect on the amount of torque loss. 45

Based on the aforementioned studies, the internal and marginal fit of titanium base abutments had comparable outcomes with other fabrication techniques. However, the cement-retained restorations using titanium base abutments could have a better fit and less generated stress than the screw-retained restorations.

### Clinical Performance

The marginal bone loss around dental implants has been proven to be one of the biological complications that can lead to implant failure. Excess cement has been suggested to have a remarkable effect on marginal bone loss. 56 One of the advantages of using titanium bases is their ability to cement the superstructure materials to themselves extraorally and to remove excess cement, thus aiding in the stabilization of the marginal bone level and reduction of the biological complications. In addition, titanium bases, as previously discussed, can withstand high occlusal forces because of their high bending moments. Thus, they can be a viable option for clinical application.

Owing to the recent introduction of titanium base abutments, few clinical studies have been conducted to assess their performance with regard to the survival and failure rates, technical and biological complications, and peri-implant soft tissue response. 57 58 59 60 61 62 63 In a prospective clinical trial, Joda et al 57 restored 44 subjects in two visits each with 50 screw-retained monolithic lithium disilicate crowns cemented extraorally to titanium bases. Most of the restorations were placed in the premolar and molar areas in both the maxillary and mandibular arches. A 2-year follow-up period revealed that the survival rate was 100% for all implants and that no biological or technical complications were recorded. 57 In a retrospective study, 42 two-piece zirconia abutments were fabricated for 27 subjects and bonded to titanium inserts. 61 All abutments were restored with final restorations, including crowns, splinted crowns, and fixed partial dentures. After 6.6 years of follow-up, seven zirconia abutments failed, mainly in the molar area, thus suggesting that zirconia abutments bonded to titanium inserts could be limited to the anterior and premolar areas. 61 A clinical report assessed the clinical performance of 24 two-piece veneered zirconia restorations cemented to titanium bases for a period of 1 year. 62 An insignificant effect was observed regarding the crestal bone level, whereas pocket depth and bleeding on probing changed significantly. A 95.8% survival rate was recorded because of the loss of one implant. Four technical complications occurred, including ceramic chipping and screw loosening, thus resulting in an 83.3% success rate of the restorations. 62

In a prospective clinical trial, Pamato et al 58 compared two groups of implant-supported crowns delivered to 21 subjects. The tested group included implants restored with 28 titanium base abutments, while the control group included implants restored with 24 cement-retained abutments. No significant difference was found between the two groups regarding bleeding on probing, pocket depth, and the mesial and distal crestal bone levels at 6-month and 1-year evaluations. The study showed that both clinical techniques were comparable, as they did not have a negative effect on the peri-implant soft and hard tissue parameters. 58 Linkevicius et al 63 assessed the level of marginal bone loss in three groups, including 2 mm or less, 2.5 mm, and 3 mm or more of vertical mucosal thicknesses. A total of 55 regular diameter implants were placed in 55 subjects and restored with monolithic lithium disilicate crowns using titanium bases. A 1-year follow-up showed that a significant marginal bone loss was recorded in the 2 mm (1.25 ± 0.8 mm) and 2.5 mm (0.98 ± 0.06 mm) mucosal thickness groups compared with the 3 mm (0.43 ± 0.37 mm) group, indicating that the vertical mucosal thickness greatly affected the marginal bone level. 63

In a recent study, the infiltration of immune cells to the peri-implant soft tissue was examined after loading implants with different types of abutments, including gold alloy, titanium, zirconia, and titanium base. 60 A total of 17 patients received 20 implants in the posterior segments of the maxillary and mandibular arches. Eight weeks later, the abutments with 1 mm peri-implant soft tissues were removed and examined. The results showed that gold alloy abutments had a significant increase in infiltration of inflammatory cells, such as macrophages, T-cells, and B-cells, whereas other abutments, including titanium base, presented insignificant changes in the inflammatory cell count. 60

Some manufacturers provide titanium base abutments with different sulcular heights to compensate for implant placement in different depth levels and variation of soft tissue heights. Multiple clinical reports have demonstrated the ability to design and fabricate ceramic abutments and crowns using titanium base to achieve the optimum emergence profile and improve the esthetic outcomes. 64 65 66 Martínez-Rus et al 65 assessed clinically the impact of different abutments and soft tissue thickness on the optical properties of lithium disilicate implant single crowns. Twenty patients were recruited in this study where 17 had thin (≤ 2 mm) and 3 had thick (> 2 mm) soft tissue thickness. Zirconia cemented to titanium base, pink-anodized titanium, gold-anodized titanium, and titanium abutments were customized using CAD/CAM technology to replicate the emergence profile of all abutments. Color change measurements were obtained 1 mm apical to the gingival margin and at the middle third of the crowns and compared with the contralateral natural tooth. They found that zirconia abutments cemented to titanium base had the lowest color change values at the measurement areas and the gingival biotype had insignificant impact on the color change of the peri-implant soft tissue with zirconia and gold-anodized abutments only. 65

Although the number of clinical studies assessing the clinical performance of titanium base abutments is limited, the use of these abutments can be considered a feasible treatment option. However, long-term clinical studies are recommended.

## Conclusion

This review was conducted to expand the knowledge about the mechanical and clinical performances of titanium base abutments. These abutments presented satisfactory mechanical properties and promising clinical behavior. Owing to the recent introduction of these abutments into dentistry, only a few clinical studies have been reported. Nevertheless, titanium bases can be employed as an alternative option to conventional approaches for restoring dental implants.
